# Enhanced Monofocal Intraocular Lenses: A Retrospective, Comparative Study between Three Different Models

**DOI:** 10.3390/jcm12103588

**Published:** 2023-05-21

**Authors:** Rita Mencucci, Alberto Morelli, Michela Cennamo, Anna Maria Roszkowska, Eleonora Favuzza

**Affiliations:** 1 Eye Clinic, Careggi Hospital, Department of Neurosciences, Psychology, Pharmacology and Child Health (NEUROFARBA), University of Florence, 50134 Florence, Italy; albertomorelli15@gmail.com (A.M.); michelacennamo@libero.it (M.C.); elefavuzza@gmail.com (E.F.); 2 Ophthalmology Clinic, Department of Biomedical Sciences, University of Messina, 98100 Messina, Italy; anna.roszkowska@unime.it

**Keywords:** enhanced monofocal, monofocal IOL, monofocal intraocular lens, enhanced monofocal iol, intraocular lens, cataract, cataract surgery, presbyopia

## Abstract

The purpose of this study was to compare the visual performance and optical quality between three new enhanced monofocal intraocular lenses (IOLs). This retrospective study included patients affected by cataracts with corneal astigmatism less than 0.75 D and no ocular comorbidities who underwent cataract surgery with bilateral implantation of Tecnis Eyhance ICB00 (Johnson & Johnson Vision Care, Inc., Jacksonville, FL, USA), Vivinex Impress XY1-EM (Hoya Surgical Optics, Singapore) or IsoPure 123 (PhysIOL, Liege, Belgium) IOLs. Three months postoperatively, monocular and binocular uncorrected and corrected distant, and intermediate and near visual acuities were measured. Binocular defocus curve, photopic contrast sensitivity, Point Spread Function (PSF), low order aberrations (LOAs), high order aberrations (HOAs), objective scatter index (OSI), halo and glare perception were also evaluated. This study included a total of 72 eyes from 36 patients. Visual acuity outcomes, PSF, LOAs, HOAs and OSI were similar between groups. There were no statistically significant differences in terms of photopic contrast sensitivity, halo or glare perception. In patients without ocular comorbidities, the Eyhance ICB00 IOL, the Vivinex Impress IOL and the Isopure IOL—even though based on different optical properties—provided similar results in terms of visual acuity, contrast sensitivity and intraocular aberrations, with no influence on photic phenomena.

## 1. Introduction

Cataracts are one of the major causes of visual impairment and blindness worldwide [1]. Modern cataract surgery with intraocular lens (IOL) implantation represents the gold standard treatment. Conventional monofocal IOLs are still the most used IOLs today [2]; these IOLs usually provide excellent uncorrected visual acuity for a fixed focus (e.g., for distant), while the patient may achieve good intermediate and near visual acuity with the help of only glasses [3].

However, in developed countries, patients expect excellent visual performance not only for distant vision, but also for intermediate and near distances due to the many daily tasks that require this range of vision (tablet and smartphone reading, driving, engaging in conversation); this is the reason why most manufacturers have introduced the concept and technology of multifocal IOLs, trying to provide a full range of focus. These IOLs may lack contrast sensitivity and may provide visual disturbances. Extended Depth of Focus (EDOF) IOLs were later released on the market, providing good distant and intermediate visual acuity with fewer visual side effects [4]. Recently, a new type of IOL called enhanced monofocal IOLs has been introduced, with the aim of providing excellent visual acuity for distant (the same as conventional monofocal lenses) while improving intermediate distant performance, without the side effects traditionally caused by multifocality [5].

The purpose of this study was to compare visual outcomes at three postoperative months of three new enhanced monofocal IOLs—the Tecnis Eyhance ICB00 (Johnson & Johnson Vision Care, Santa Ana, CA, USA), the Vivinex Impress XY1-EM (Hoya Surgical Optics, Singapore) and the Isopure 123 IOLs (PhysIOL, Liege, Belgium)—in terms of distant, intermediate and near visual acuities, refractive outcomes, contrast sensitivity, objective ocular optical quality and halo and glare perception.

## 2. Materials and Methods

### 2.1. Study Design

In this retrospective single-center nonrandomized comparative series of cases, the records of patients who had undergone cataract surgery with bilateral implantation of one of the following enhanced monofocal IOLs were reviewed: the Tecnis Eyhance ICB00, the Hoya Vivinex Impress XY1-EM or the PhysIOL Isopure 123 IOL. 

The study was conducted at Careggi Eye Hospital in Florence, Italy between May and December 2022, following the tenets of the Declaration of Helsinki. Informed consent was obtained from all subjects involved in the study.

The inclusion criteria was comprised of adult patients (>18 years) who had undergone uncomplicated consecutive bilateral cataract surgery with bilateral implantation of one of the three IOL models in the capsular bag, with a follow-up of at least three months and a complete ophthalmological assessment performed three months after surgery, including defocus curve, aberrometry, contrast sensitivity and objective evaluation of the ocular optical quality; age more than 18 years; and corneal astigmatism less than 0.75 D.

The exclusion criteria included pregnant women, amblyopia, history of ocular surgery other than cataract surgery (including corneal or refractive surgery), axial length over 25.0 mm or less than 18.0 mm, keratoconus, pellucid marginal degeneration, pterygium, corneal endothelial dystrophy, chronic or recurrent uveitis, acute ocular disease or external/internal infection, diabetes mellitus with retinal changes, retinal vasculopathy, glaucoma or ocular hypertension under treatment, pathological miosis or pseudoexfoliation syndrome.

### 2.2. Patient Evaluations

At the baseline visit, before surgery, all patients underwent a comprehensive evaluation that included the measurement of the monocular and binocular uncorrected distance (4 m) visual acuity (UDVA) and corrected distance visual acuity (CDVA) under photopic conditions (85 cd/m^2^) and 100% contrast with Early Treatment Diabetic Retinopathy Study (ETDRS) charts, subjective and objective refraction, biomicroscopy, Goldmann applanation tonometry, optical biometry (IOLMaster 500, Carl Zeiss Meditec AG, Jena, Germany), corneal topography (Sirius, CSO Costruzione Strumenti Oftalmici, Scandicci, Italy), dilated fundoscopy and macular optical coherence tomography (DRI OCT Triton 3D, Topcon Medical Systems, Inc., Paramus, NJ, USA). The IOL power and predicted postoperative refraction were based on biometry data measured using the IOLMaster 500 biometry device and calculated using the Barrett Universal II formula [6]. The IOL power was chosen to obtain the predicted postoperative refraction closest to emmetropia for both eyes. 

After surgery, patients were evaluated at 1 day, 1 week, 1 month and 3 months. 

At the three-month follow-up, in addition to the slit lamp examination, tonometry, refraction, UDVA and CDVA, uncorrected intermediate (66 cm) visual acuity (UIVA), distance-corrected intermediate visual acuity (DCIVA), uncorrected near (40 cm) visual acuity (UNVA) and distance-corrected near visual acuity (DCNVA) were measured using high-contrast ETDRS printed charts (Precision Vision) under photopic conditions. Moreover, binocular defocus curves were obtained using the best distance correction, with the progressive addition of 0.50 D increments (range +2.00 to −2.50 D. The binocular contrast sensitivity was assessed under photopic conditions (85 cd/m^2^) using the CSO Vision Chart (CSO Costruzione Strumenti Oftalmici).

All patients underwent an aberrometric evaluation under scotopic light of 3 cd/m^2^ using a new pyramidal aberrometer: Osiris-T (CSO, Costruzione Strumenti Oftalmici). Intraocular high order aberration (HOA) root mean square (RMS), intraocular low-order aberration (LOA) RMS and the intraocular spheric aberration (SA) RMS, as well as the Point Spread Function (PSF) Strehl Ratio were obtained for a 4 mm pupil. The PSF describes the quality response of an imaging system and is expressed by the Strehl ratio, with 1 indicating a perfect optical system. The Strehl ratio is the ratio between the intensity of the real PSF and the intensity of the diffraction-limited PSF [7].

Furthermore, an objective evaluation of the ocular optical quality was performed using an AcuTarget HD Analyzer (Visiometrics Inc., Costa Mesa, CA, USA)—an Optical Quality Assessment System (OQAS) product based on double-pass aberrometer technology. The device was used at a 4.0 mm pupil to obtain the objective scatter index (OSI), which indicates the intraocular scatter light that is computed by evaluating the amount of light on the periphery of the double-pass image in relation to the amount of light on the center.

The subjective experience of halos and glare evaluated at the three-month follow-up using Items 17 and 38 of the National Eye Institute-Refractive Error Quality of Life-42 (NEI-RQL-42) questionnaire [8] was recorded.

### 2.3. Surgery

All the surgical interventions were performed by the same expert surgeon (R.M.) using topical anesthesia. After the creation of a 2.2 mm temporal corneal incision and a continuous capsulorhexis of ~5.5 mm, the phacoemulsification was performed by the stop-and-chop technique. IOLs were implanted into the capsular bag, and at the end of surgery, the incisions were sealed with hydrosutures after the intracameral cefuroxime was injected into the anterior chamber. Each patient followed a postoperative treatment plan with topical nonsteroidal anti-inflammatory drugs for 1 month, 2 times daily and steroid plus antibiotic drops 4 times daily for the first 2 weeks.

### 2.4. Intraocular Lenses

The Tecnis Eyhance ICB00 (Johnson & Johnson Vision Care, Santa Ana, CA, USA) is the first enhanced monofocal lens to be introduced in cataract surgery. This is a single-piece foldable hydrophobic monofocal IOL with the same features as the respective standard monofocal ZCB00—except for its modified aspheric anterior surface, which can create a continuous power profile from the periphery to the center, providing a stretch in the range of vision and improving vision for intermediate tasks [9]. According to the recent case series of Mencucci et al. [10], this IOL was shown to provide satisfactory intermediate distance spectacle independence, while preserving the distance visual acuity performance of the standard monofocal ZCB00. 

The Vivinex impress XY1-EM (Hoya Surgical Optics, Singapore) is a new single-piece enhanced monofocal IOL made of glistening-free hydrophobic acrylate with an aspherical optic, which provides an extended depth of field to support an intermediate range. It has a combination standard aspheric and even-order aspheric surface with microscopic angulations. No studies are available in the literature investigating the clinical properties of this enhanced monofocal lens. 

The IsoPure 123 IOL (PhysIOL, Liege, Belgium) is a foldable, single-piece acrylic hydrophobic glistening-free lens with four closed haptics; it has an ultraviolet and light-blue filter. The Isopure IOL is a monofocal lens that combines an anterior and posterior surface profile of increased negative spherical aberration and is fine-tuned for each diopter on the whole. According to the recent paper of Bova et al. [11], this lens may provide a good solution for distant and intermediate vision with minimal impact on contrast sensitivity, without increasing optical aberrations or causing any photic symptoms.

### 2.5. Statistical Analysis

Data analysis was performed using SPSS Statistics for Windows software (version 25.0, IBM Corp.). The normal distribution of the variables was checked by using the Shapiro–Wilk test of normality. Collected data were examined and presented using descriptive analysis. Continuous variables were presented as a mean ± standard deviation (SD). To obtain a statistical comparison of the three groups, an analysis of the continuous variables was performed using the one-way ANOVA test combined with the Tukey post hoc test. For all statistical tests, a *p*-value less than 0.05 was considered statistically significant and 95% confidence intervals were considered.

## 3. Results

The study included 72 eyes from 36 patients who had undergone bilateral cataract surgery. Patients were retrospectively divided into three groups according to the IOL implanted: the Eyhance group (12 patients, 24 eyes) included eyes implanted with Tecnis Eyhance lenses, the Impress group (12 patients, 24 eyes) included eyes implanted with Hoya Impress lenses and the Isopure group (12 patients, 24 eyes) included eyes implanted with PhysIOL Isopure lenses. All surgeries were uneventful and no adverse events were observed during the follow-up period.

### 3.1. Preoperative Data

Table 1 shows the preoperative data of the three groups. No significant difference was observed between the groups (all *p* > 0.05, ANOVA test).

### 3.2. Visual and Refractive Outcomes

Postoperative visual outcomes at three months are shown in Table 2.

All patients reached high levels of monocular and binocular uncorrected distance visual acuity (UDVA). 

Monocular and binocular uncorrected intermediate visual acuity (UIVA) were similar between the three groups (*p* = 0.146 and *p* = 0.488, respectively). The best corrected intermediate visual acuity was obtained in the three groups with a spherical addition of 1.29 D ± 0.59 D in the Eyhance group, 1.42 D ± 0.35 D in the Impress group and 1.33 D ± 0.32 D in the Isopure group (*p* = 0.602). 

All participants showed similar monocular and binocular uncorrected near visual acuity (UNVA, *p*= 0.453 and *p* = 0.385, respectively). The mean addition needed for near visual acuity was 2.35 ± 0.70 D in the Eyhance group, 2.54 ± 0.50 D in the Impress group and 2.67 ± 0.48 D in the Isopure group, with no significant differences between the groups (*p* = 0.150).

The objective spherical equivalent (SE) measured three months after surgery was −0.34 D ± 0.26 D in the Eyhance group, −0.35 D ± 0.25 D in the Impress group and −0.52 D ± 0.42 D in the Isopure group, with no significant differences between the groups (*p* = 0.111).

### 3.3. Binocular Defocus Curve

**Figure 1:**
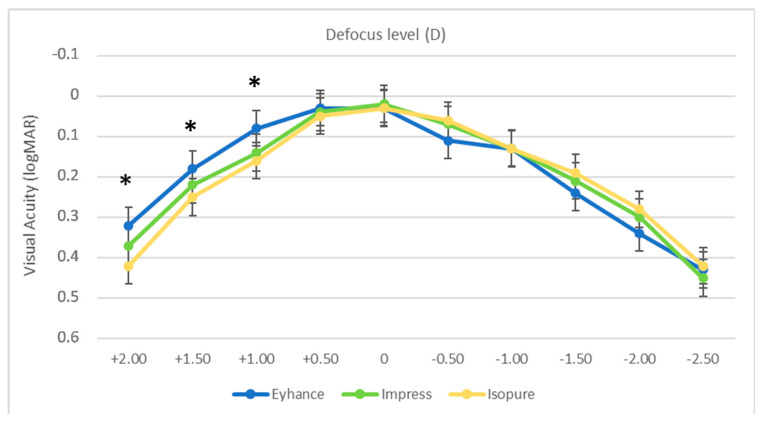
Mean binocular defocus curves in the 3 intraocular lens groups (logMAR = logarithm of the minimum angle of resolution; D = diopters). * *p* < 0.05 Eyhance vs. Isopure.

Figure 1 reports the binocular defocus curve measured in the three groups, obtained in the range of +2.00 D to −2.50 D with 0.5 D incremental steps. The three curves had a similar profile, showing a peak at defocus 0.00 D (4 m), with a gradual decrease in visual acuity with positive and negative defocus.

A statistically significant difference could be observed only between the Eyhance and Isopure groups for the +2.00 D, +1.50 D and +1.00 D defocus levels (*p* = 0.045, *p* = 0.036 and *p* = 0.019, respectively). 

Better, but not statistically significantly (*p* > 0.05), results could be observed for the −0.50 D, −1.50 D and −2.00 D defocus levels in the Impress and Isopure groups compared to the Eyhance group. 

### 3.4. Optical Quality, Contrast Sensitivity and Halo and Glare Perception

Optical quality parameters measured using the Osiris-T pyramidal aberrometer and the AcuTarget double-pass aberrometer are shown in Table 3. Assessments were taken for a 4.0 pupil diameter. The Point Spread Function (PSF), intraocular Low Order Aberrations (LOA), intraocular High Order Aberration (HOA), intraocular Spherical Aberration (SA) and Ocular Scatter Index (OSI) were similar between the groups (*p* = 0.184, *p* = 0.108, *p* = 0.092, *p* = 0.147 and *p* = 0.544, respectively).

**Figure 2:**
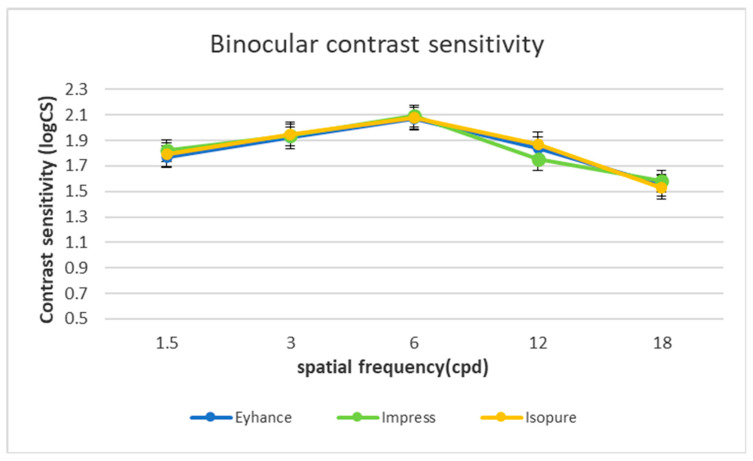
Mean photopic binocular contrast sensitivity.

Mean photopic binocular contrast sensitivity measurements are reported in Figure 2. Differences were not statistically significant (*p* > 0.05).

The results of Items 17 and 38 of the NEI-RQL-42 questionnaire concerning photic phenomena were very satisfying in all three groups, as all participants declared the total absence of glare or halo perception at three months post-surgery.

## 4. Discussion

Cataracts represent a major cause of visual and psychological impairment, affecting quality of life—especially for elderly patients [1]. Standard cataract surgery has high success rates with conventional monofocal IOLs, even though they provide excellent visual acuity only for a fixed focus distance [3]. Daily tasks have changed, and they now also require good vision for the intermediate range of focus; as such, manufacturers have studied new solutions to overcome this problem. Accommodative, bifocal, trifocal, and EDOF IOLs have been introduced into the market to offer a higher spectacle independence compared to conventional monofocal IOLs. However, the potential drawbacks of these IOLs are their higher cost, the reduction of optical performance in low-light conditions, decreases in visual quality and the perception of photic phenomena such as haloes and glare [3]. Hence, the continuous work to reshape IOL optical quality to overcome these issues has led to the design of new, enhanced monofocal IOLs. 

The first enhanced monofocal IOL available for cataract surgeons was the Eyhance ICB00 IOL. Several studies showed better intermediate and similar distant visual acuity with no impairment of optical quality or contrast sensitivity compared to conventional monofocal IOLs [10,12,13,14]. Moreover, a metanalysis by Wan et al. [15] including 680 eyes implanted with Eyhance IOLs recently confirmed these findings. 

Fewer studies investigating the visual performance and optical quality of the enhanced monofocal IOL Isopure 123 are present in the literature. Stodulka et al. [16] recently investigated the visual performance of the Isopure lens, showing excellent distant and intermediate visual acuity with good tolerance of residual refractive cylinders along with high contrast sensitivity. The study of Bova et al. [11] investigated the visual and aberrometric outcomes of the Isopure 123 lens, showing a significantly higher intermediate visual acuity compared to a conventional monofocal IOL, without increasing optical aberrations or photic phenomena. A recent study by Bernabeu-Arias et al. [17] found excellent visual performance for far vision and functional intermediate vision, with an extended range of vision. In the retrospective study of Lesieur et al. [18], which compared the outcomes of the Isopure 123 IOL, the Synthesis+ (Cutting Edge, France) and the Lucidis (SAV-IOL, Swiss), the Isopure lens obtained visual outcomes similar to those presented in this paper. A mini-monovision approach showed even better intermediate and near visual outcomes [19].

Given that the Vivinex Impress lens has just recently been released, there have been no clinical studies published in the literature. A recent paper of Pieh et al. [20] investigated the optical properties at the optical bench of the Impress IOL, showing a similar light distribution for the intermediate range compared to the Isopure lens. 

This is the first clinical study to compare the visual outcomes, contrast sensitivity and aberrometric parameters of these three different models of enhanced monofocal IOLs. 

Excellent uncorrected distant and corrected intermediate and near visual acuity were obtained in all the participants, with no statistically significant differences between the groups—showing results similar to those previously published in the literature [10,11,12,17]. 

In our study, the uncorrected intermediate visual acuity of the three groups was similar and were consistent with the published studies of Mencucci et al. [10], Bova et al. [11], Bernabeu-Arias et al. [17] and Lesieur et al. [18]. 

The defocus curve profile was similar in the three groups, even though in the Isopure group, slightly poorer outcomes than Eyhance for the +2.00 D to +1.00 D range could be observed; however, these findings are of little clinical interest as they concern the positive defocus range. In the myopic defocus range, the three lenses behaved similarly and the Isopure and Impress IOLs showed slightly better (even if not statistically significant) results for the −0.50 D, −1.50 and −2.00 D defocus levels. In the study of Lesieur et al. [18], the defocus curve of the Isopure lens was slightly worse than the curve obtained in our study. 

In the present study, the three IOLs showed excellent results in terms of PSF, LOA, HOA and OSI. We also investigated the contribution of spherical aberration, which is known to cause substantial optical quality degradation and loss of contrast sensitivity. Eyhance, Impress and Isopure lenses provided similar outcomes measurements for the above-mentioned parameters, related to the good contrast sensitivity results and to the total absence of halo and glare perception in the three groups. 

The limitations of our work were the retrospective nature of the study, the low number of patients and the limited follow-up of three months. Moreover, it would be interesting to compare the outcomes of a conventional monofocal IOL with the results obtained with the three IOLs evaluated in the present work. 

Further prospective randomized studies are warranted to assess the visual outcomes of the different types of IOLs and to provide useful tools for surgeons to determine the best candidate patient for each type of IOL.

In conclusion, the Tecnis Eyhance, the Hoya Vivinex Impress and the PhysIOL Isopure enhanced monofocal IOLs—even though based on different optical profiles—showed similar results in terms of visual outcomes, aberrometry and photic phenomena perception. They may represent an interesting option in the standard treatment of cataracts because they provide good spectacle independence for intermediate distances, while preserving excellent performance for distance vision.

## Figures and Tables

**Table 1:** Preoperative data of enrolled patients.

		Mean	Standard Deviation	*p*-Value (ANOVA)
**Age** **(years)**	**Eyhance**	79.38	5.123	
	**Impress**	78.04	4.582	
	**Isopure**	81.79	4.452	0.187
**UDVA** (logMAR)	**Eyhance**	0.463	0.128	
	**Impress**	0.454	0.204	
	**Isopure**	0.404	0.120	0.400
**CDVA** (logMAR)	**Eyhance**	0.396	0.120	
	**Impress**	0.396	0.123	
	**Isopure**	0.375	0.129	0.831
**SE** (D)	**Eyhance**	1.563	0.785	
	**Impress**	1.208	0.729	
	**Isopure**	1.206	1.028	0.326
**CYL** (D)	**Eyhance**	0.792	0.252	
	**Impress**	0.750	0.489	
	**Isopure**	0.667	0.493	0.570
**AL** (mm)	**Eyhance**	23.602	1.157	
	**Impress**	24.258	0.652	
	**Isopure**	23.377	0.705	0.644

UDVA (Uncorrected Distant Visual Acuity), CDVA (Corrected Distant Visual Acuity), SE (spherical equivalent), CYL (cylinder), AL (axial length).

**Table 2:** Three-month visual outcomes in the three intraocular lens groups.

	Eyhance	Impress	Isopure	*p*-Value (ANOVA)
**UDVA MON**	0.041 ± 0.042	0.033 ± 0.044	0.04 ± 0.05	0.839
**UDVA BIN**	0.03 ± 0.04	0.02 ± 0.04	0.03 ± 0.04	0.794
**BCDVA MON**	0.03 ± 0.04	0.03 ± 0.04	0.02 ± 0.04	0.589
**BCDVA BIN**	0.017 ± 0.038	0.021 ± 0.038	0.021 ± 0.041	0.915
**UIVA MON**	0.283 ± 0.096	0.233 ± 0.117	0.225 ± 0.115	0.146
**UIVA BIN**	0.142 ± 0.065	0.133 ± 0.048	0.158 ± 0.097	0.488
**DCIVA MON**	0.231 ± 0.074	0.222 ± 0.035	0.210 ± 0.085	0.399
**DCIVA BIN**	0.136 ± 0.073	0.110 ± 0.035	0.129 ±0.076	0.278
**BCIVA MON**	0.071 ± 0.086	0.058 ± 0.075	0.050 ± 0.051	0.603
**BCIVA BIN**	0.035 ± 0.047	0.042 ± 0.062	0.025 ± 0.044	0.535
**UNVA MON**	0.475 ± 0.119	0.446 ± 0.102	0.438 ± 0.107	0.453
**UNVA BIN**	0.337 ± 0.042	0.363 ± 0.071	0.354 ± 0.072	0.385
**DCNVA MON**	0.457 ±0.115	0.433 ±0.067	0.408 ± 0.090	0.498
**DCNVA BIN**	0.291 ± 0.067	0.301 ± 0.081	0.316 ±0.092	0.378
**BCNVA MON**	0.088 ± 0.112	0.067 ± 0.076	0.058 ± 0.041	0.446
**BCNVA BIN**	0.033 ± 0.048	0.054 ± 0.072	0.058 ± 0.054	0.298

Data are shown in logMAR (logarithm of the minimum angle of resolution), UDVA (Uncorrected Distant Visual Acuity), BCDVA (Best Corrected Distant Visual Acuity), UIVA (Uncorrected Intermediate visual acuity), DCIVA (distant-corrected intermediate visual acuity), BCIVA (Best Corrected Intermediate visual acuity), UNVA (Uncorrected Near Visual Acuity), DCNVA (distant-corrected near visual acuity), BCNVA (Best Corrected Near Visual Acuity), MON (Monocular), BIN (Binocular).

**Table 3:** Three-month ocular optical quality parameters at a 4.0 pupil diameter.

	Eyhance	Impress	Isopure	*p*-Value (ANOVA)
**PSF**	0.189 ± 0.041	0.211 ± 0.049	0.193 ± 0.042	0.184
**LOA**	0.293 ± 0.098	0.293 ± 0.098	0.238 ± 0.110	0.108
**HOA**	0.177 ± 0.045	0.202 ± 0.074	0.166 ± 0.047	0.092
**SA**	0.078 ± 0.038	0.067 ± 0.024	0.060 ± 0.035	0.147
**OSI**	1.375 ± 0.527	1.433 ± 0.346	1.287 ± 0.485	0.544

PSF (Point Spread Function), LOA (Low Order Aberrations), HOA (High Order Aberration), SA (Spherical Aberration) and OSI (Ocular Scatter Index).

## Data Availability

Data not publicly available.
